# Research on the design and mechanical properties of extended constant resistance energy-absorbing anchor bolts

**DOI:** 10.1371/journal.pone.0353592

**Published:** 2026-10-27

**Authors:** Shiyang Cheng, Zhi Tang, Shengrui Zhang, Mingqiu Yu

**Affiliations:** 1 College of Mechanics and Engineering, Liaoning Technical University, Fuxin, China; 2 Institute of Disaster Rock Mechanics, Liaoning University, Shenyang, China; China Construction Fourth Engineering Division Corp. Ltd, CHINA

## Abstract

To address the premature fracture of anchor bolts caused by the mismatched yielding distance between anchor bolts and hydraulic supports in the advanced support section of rock burst-prone roadways, an elongated constant-resistance energy-absorbing anchor bolt was designed—with its yielding distance specifically coordinated to match that of hydraulic supports. ABAQUS numerical simulation was utilized to investigate the mechanical characteristics of this novel anchor bolt under static loading, as well as under varying impact energies and impact velocities. A comparative analysis was further performed against the mechanical performance of conventional anchor bolts. The results reveal the following: Under static loading, the elongated constant-resistance energy-absorbing anchor bolt exhibits a fracture load of 160.6 kN, a fracture displacement of 523.2 mm, an elongation rate of 20.93%, and an energy absorption capacity of 69.7 kJ. Under impact velocities within 9 m/s, the impact velocity has little effect on the fracture load, elongation, and energy absorption of constant-resistance energy-absorbing bolts, while the impact resistance time exhibits a nonlinear decreasing trend with increasing impact velocity. Under both static and dynamic loading conditions, the extended constant-resistance energy-absorbing bolt exhibits superior impact resistance and can effectively address the problem of premature bolt fracture caused by the mismatch between the bolt yielding distance and that of the hydraulic support in the three-level energy-absorbing support system. The findings provide a new perspective for bolt design.

## 1. Introduction

Rock burst is one of the major dynamic hazards encountered in deep coal mining operations in China [1,2], with its occurrence frequency increasing significantly with mining depth [3,4]. Under the combined effects of deep-seated high stress and intense mining activities, the risk of tunnel rock mass instability has significantly increased, posing a serious threat to mining safety [5].

Currently, many scholars have achieved some research results in the field of roadway support and stability control. Wang Aiwen et al. [6–8] analyzed rock burst phenomena in roadways supported by rock bolts based on instability theory. They investigated the influence of bolt spacing on the critical resistance zone, critical load, and released energy during rock burst occurrence. Li Qinghai et al. [9] addressed the phenomenon of anchor preload loss in rock masses under frequent dynamic loading impacts. They analyzed the evolution trend of anchor preload under dynamic loading conditions and investigated the influence patterns of dynamic loading parameters on anchor performance. Meng Qingbin et al. [10] revealed the deformation patterns of the “shotcrete + anchor cables/U-shaped steel supports + grouting” support unit during different support phases. Yiqun Zhou [11] analyzed the distribution characteristics of prestress fields in bolted support under different anchor rock formations and anchor lengths. Chun Zhu et al. [12] investigated the influence patterns of design parameters for different constant-resistance device geometries on the static and dynamic performance of NPR rock bolts.

Scholars have achieved significant results in the field of tunnel support systems. However, existing support systems exhibit insufficient spatial coordination in rock mass deformation, limited control range for active support, inadequate pressure-relieving capacity for passive support, and a lack of synergistic control between the two [13]. Within the framework of the current three-level energy-absorbing support system [14,15], a pronounced mismatch in yield distance is observed between the energy-absorbing bolts and the energy-absorbing hydraulic supports. When a rockburst occurs, the surrounding rock experiences intense deformation. The hydraulic support, benefiting from its long yield stroke, is able to sustain supporting resistance throughout the process of roof subsidence. The conventional energy-absorbing bolt, however, possesses a considerably shorter yield distance and thus reaches or approaches its ultimate deformation limit before the surrounding rock deformation is fully accommodated. As a result, the bolt stress rises rapidly to the breaking load, leading to brittle fracture. This temporal incompatibility in deformation precludes effective collaborative load transfer between the bolts and the hydraulic supports in both time and space, ultimately undermining the energy-dissipation capacity and impact resistance of the entire three-level support system.

Therefore, an extended constant-resistance energy-absorbing anchor rod has been designed. By optimizing its structural parameters, the displacement distance is precisely matched with the energy-absorbing hydraulic support, preventing premature failure of the anchor rod during impact. Investigate the influence of static load, varying impact energy, and impact velocity on the impact resistance properties of extended constant-resistance energy-absorbing rock bolts.

## 2. Extended constant-resistance energy-absorbing anchor rod design

### 2.1. Determining the clearance distance

(1) Statistical Analysis of Surrounding Rock Deformation Characteristics

The roof of a roadway refers to the rock strata or coal seam forming its upper boundary. It bears the pressure from overlying rock masses while providing coverage and protection for the roadway’s internal space. Roof subsidence not only reflects the extent of damage caused by rock burst to roof stability but also serves as a critical indicator for assessing subsequent safety risks in mine extraction operations [16,17].

For example, at the Nalinhe No. 2 Mine in the Ordos Mining Area, during the development of the 31102 working face, secondary mining activity caused five significant dynamic phenomena in the 31102 return airway. The occurrence times and their relative positions to the working face are shown in Fig 1.

**Fig 1:**
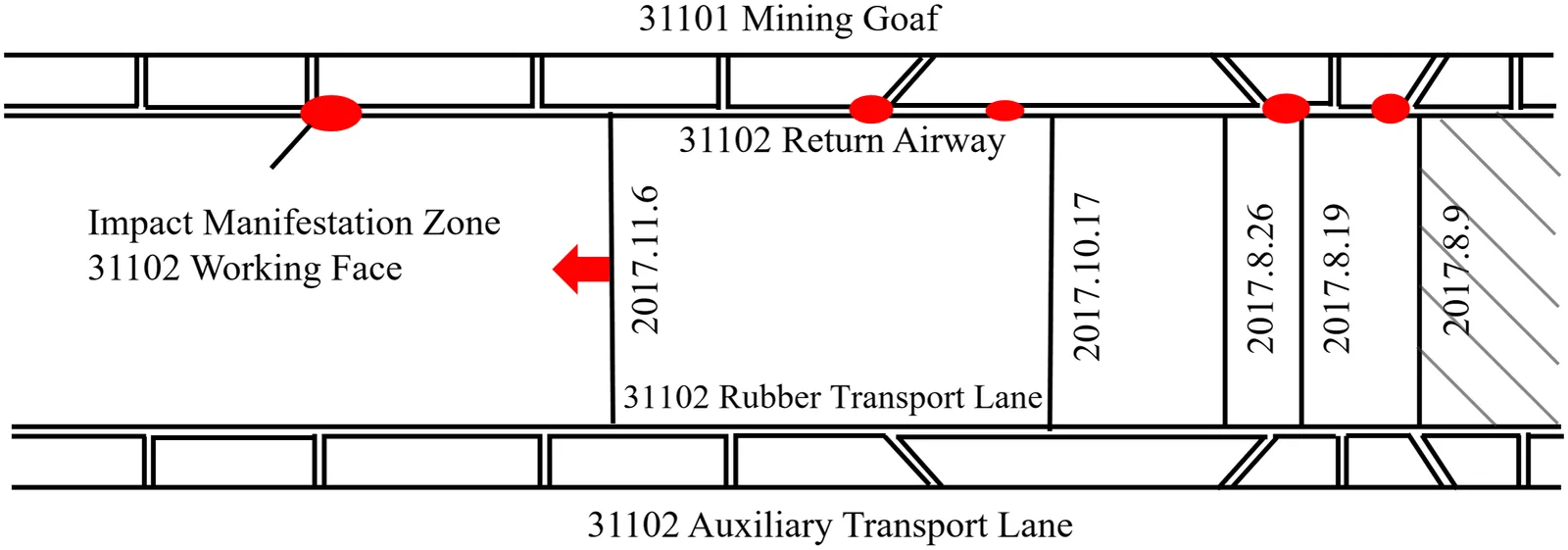
Schematic of the location of the Narin River No. 2 well power emergence.

On August 26, 2017, a dynamic manifestation disaster occurred, affecting an area up to 45 m in length. At the moment of the disaster, over 10 support props buckled and deformed, 35 roof beams developed downward plastic deformation, and the maximum roof displacement in the roadway reached 600 mm. Photographs of the affected roof are shown in Fig 2.

**Fig 2:**
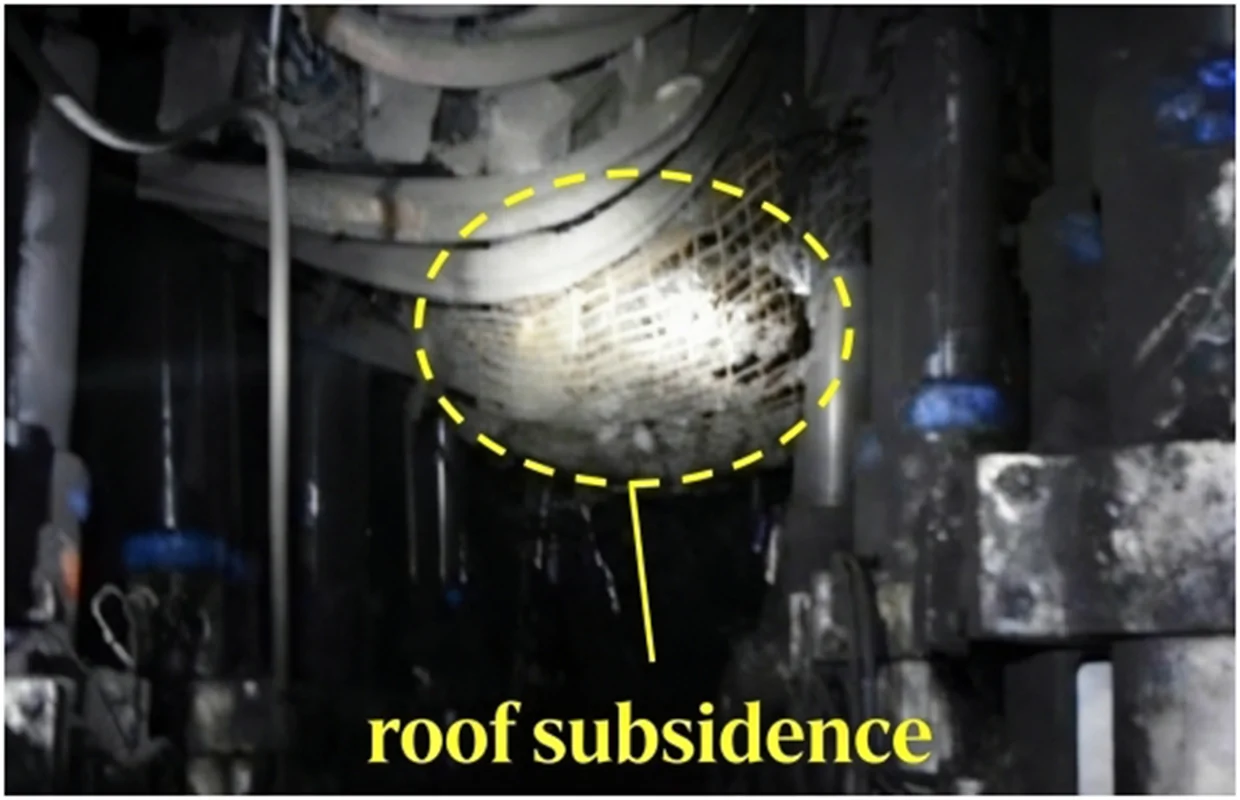
The top plate of the Narin River No. 2 shaft.

By collecting rockburst data from seven typical coal mines, including Nalinhe No. 2 Mine in the Ordos Mining Area, Laohutai Mine of Fushun Mining Group Co., Ltd., Yima Coal Mine, Hongqinghe Coal Mine, and Menkeqing Mine in the Hujierte Mining Area, a total of 28 rockburst events were recorded. The statistical results of roof subsidence are presented in Table 1. During rockburst occurrences, the roof subsidence is mainly concentrated in the range of 100–500 mm, with the 200–300 mm interval being the most typical.

**Table 1:** Statistics of roof subsidence.

Roof subsidence/mm	Occurrence count/ time	Proportion/%
≤100	4	14.28
≤200	8	28.57
≤300	6	21.43
≤400	5	17.86
≤500	4	14.28
>500	1	3.58

The deformation behavior of both sides of a roadway exerts a significant influence on mine safety production, the stability of support structures, and the formulation of subsequent mining plans. The extent of deformation along both sides not only directly reflects the mechanical response and failure patterns of the roadway’s surrounding rock under rockburst conditions but also serves as a critical quantitative indicator for assessing the mine’s rockburst hazard level and evaluating potential safety hazards.

By collecting and selecting rock burst data from six coal mines, namely Qianqiu Coal Mine, Laohutai Mine of Fushun Mining Group Co., Ltd., Yima Coal Mine, Gushan No.1 Well, Junde Coal Mine in Hegang Mining Area, and Meiyukou Mine in Datong Mining Area, a total of 35 rock burst events were accumulated. The statistical results of the deformation amounts of the two sidewalls are presented in Table 2. When rock bursts occur, the deformation of the two sidewalls is mainly concentrated in the range of 200–500 mm, indicating that the sidewalls are more prone to substantial deformation during rock bursts, and the overall deformation magnitude is slightly greater than the roof subsidence.

**Table 2:** Statistics of two-sidewall deformation.

Two-sidewall deformation interval/mm	Occurrence count/times	Proportion/%
≤100	2	5.71
≤200	11	31.43
≤300	5	14.29
≤400	8	22.86
≤500	5	14.29
>500	4	11.42

(2) Analysis of Factors Affecting Stent Retraction Distance

The clearance distance of hydraulic supports used in coal mines refers to the allowable distance of roof subsidence or relative component movement when the support is subjected to external forces such as roof pressure or rockburst. The clearance distance is influenced by numerous factors, such as:

1) Geological Conditions: In mines with soft roof rock and unstable coal seam conditions, supports must accommodate greater surrounding rock deformation, necessitating larger clearance distances. For example, in mines with shale or other soft rock roofs, support clearance distances typically range from 200 to 500 mm to prevent damage caused by excessive resistance to deformation.
2) Rockburst Factors: For mines experiencing frequent and high-intensity rockbursts, a reasonable clearance distance must be designed to absorb impact energy, typically ranging from 300 to 800 mm. During an impact event, ample clearance space allows the support structure to absorb energy through deformation, thereby minimizing catastrophic damage to roadways and equipment.
3) Support Type and Structural Factors: The clearance distance of different hydraulic support types is constrained by their inherent structures. For instance, conventional hydraulic supports have limited basic clearance (50–150 mm) due to the extension range of their hydraulic cylinders. However, when subjected to intense pressure or impact, the clearance can be extended to 300–500 mm by activating the telescoping mechanism.
4) Mining Process Factors: In mines employing fully mechanized top coal caving, the roof undergoes dynamic changes during caving. Supports must accommodate roof subsidence and collapse, with a typical clearance distance of 150–400 mm to balance effective support with pressure release requirements.

Based on the above analysis, under rockburst conditions, the effective yielding distance of hydraulic supports is mainly concentrated in the range of 100–500 mm, and in some strong rockburst mines, it can reach up to 800 mm.

(3) Determination of the Clearance Distance for Extended Constant-Resistance Energy-Absorbing Anchor Rods

Conventional bolts and some energy-absorbing bolts exhibit a fracture displacement of approximately 100–200 mm. Under rockburst conditions, however, roof subsidence ranges from 100 to 500 mm, sidewall deformation from 200 to 500 mm, and the effective yielding stroke of hydraulic supports falls within 100–500 mm. Given that the fracture displacement of conventional bolts is considerably less than both the actual surrounding-rock deformation and the support yielding distance, the bolts would reach their ultimate bearing capacity and fail before the hydraulic supports during a rockburst. This premature failure prevents the bolts from working in synergy with the hydraulic supports, thereby significantly undermining the effectiveness of the three-tier energy-absorbing support system.

Therefore, to realize the coordinated compatibility between the bolt and the yielding stroke of the hydraulic support, and to avoid premature failure of the bolt caused by inadequate yielding capacity, the yielding distance of the novel bolt should be designed to reach 500 mm.

### 2.2. Structural design of extended constant-resistance energy-absorbing anchor rods

The constant-resistance energy-absorbing bolt consists of a threaded steel rod, a bolt sleeve, an energy-absorbing tube, a bearing plate, an expanded conical sleeve, and a locking nut. The cross-section of the overall structure is shown in Fig 3.

**Fig 3:**
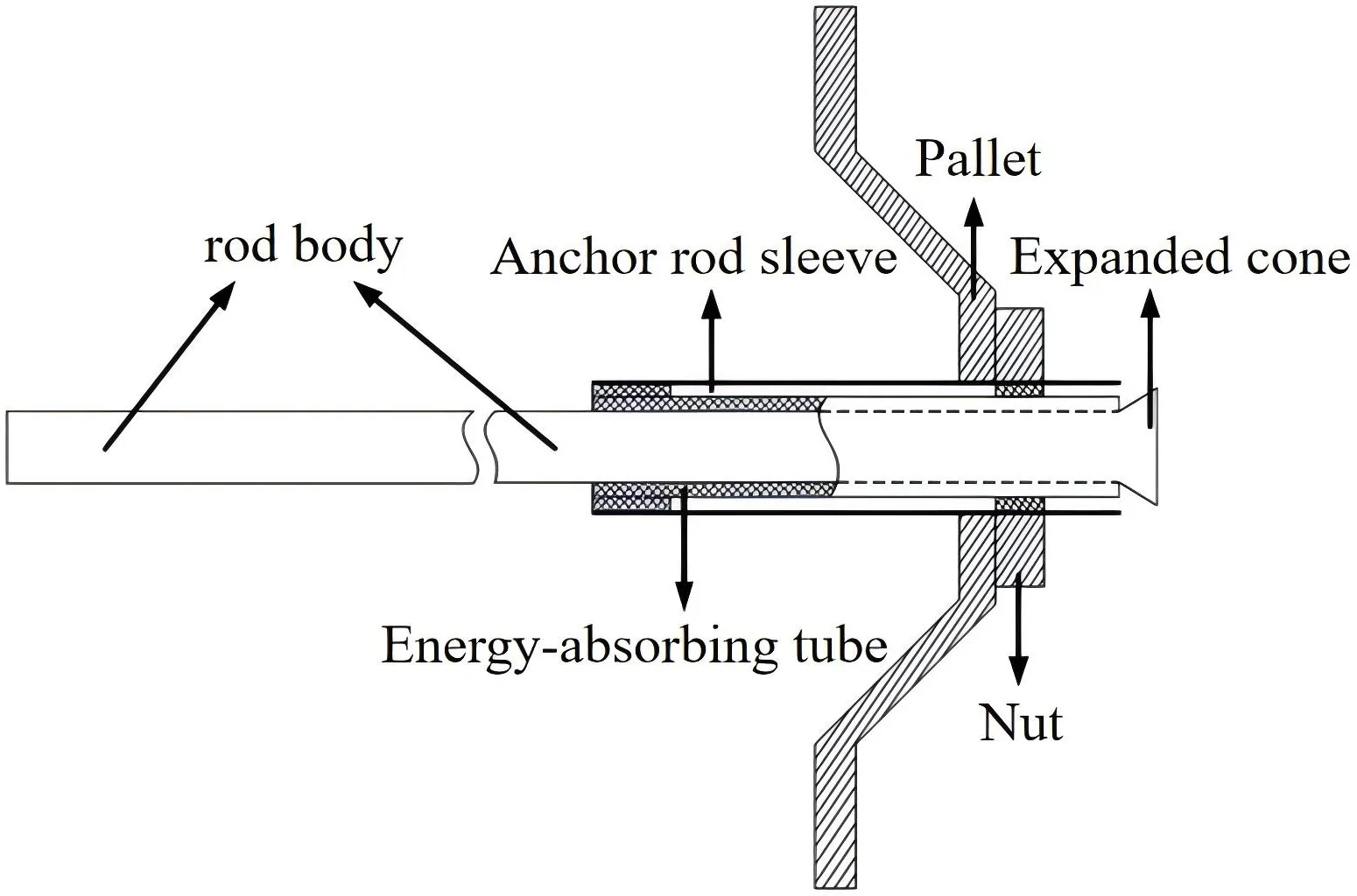
Overall cross-sectional view of the longened constant-resistance energy-absorbing bolt.

An expansion-type energy-absorbing device is employed owing to its characteristic of maintaining a constant resistance throughout the deformation process [18,19]. The energy-absorbing element is a thin-walled metal cylindrical tube, the length of which is set at 300 mm through comprehensive mechanical analyses, with reference to both the roof subsidence monitoring data obtained from rockburst-prone coal mines and the appropriate yielding stroke range of underground support systems. The expanded conical sleeve adopts a tapered transitional geometry, wherein the smaller-end diameter matches that of the bolt rod and is slightly less than the internal diameter of the energy-absorbing tube, whereas the larger-end diameter exceeds the tube’s internal diameter so as to induce stable circumferential plastic expansion of the tube under tensile loading. Positioned externally to the rod are the tube and the bolt sleeve, which are joined by welding into a single integrated assembly with their lower ends aligned flush. The lower end of the tube, featuring a straight-edged opening, rests upon the conical sleeve at the rod’s lower extremity; the two components together constitute the expanded cone–sleeve damping mechanism. A bearing plate is mounted around the bolt sleeve, and a locknut is securely fastened beneath the plate to apply a pre-tensioning force and uniformly transmit the anchorage load to the surrounding rock mass of the roadway.

### 2.3. Load-bearing capacity calculation and parameter determination of the bolt energy-absorbing device

The load-bearing capacity of the energy-absorbing device is a key parameter affecting the impact resistance performance of the bolt. If the capacity is too high, the device may fail to activate in a timely manner, and the bolt body may fracture prematurely; if the capacity is too low, the device will deform too early, losing its energy-absorbing and yielding function.

Let the outer diameter, inner diameter, and wall thickness of the energy-absorbing tube be $2R_{\text{0}}$, $2r_{\text{0}}$, and *t*, respectively. After diametral expansion deformation, the outer diameter of *t*he deformed tube equals the outer diameter of the larger end of the expanded conical sleeve, denoted as $2R_{\text{1}}$, with the corresponding inner diameter being $2r_{\text{1}}$. The cone angle of the expanded conical sleeve is α; the axial compressive stress is $\sigma_{z}$, the radial normal stress is $\sigma_{n}$, and the circumferential tensile stress is $\sigma_{\theta }$. The coefficient of friction between the inner wall of the energy-absorbing tube and the expanded conical sleeve is μ. A schematic diagram of the deformation energy absorption is shown in the Fig 4.

**Fig 4:**
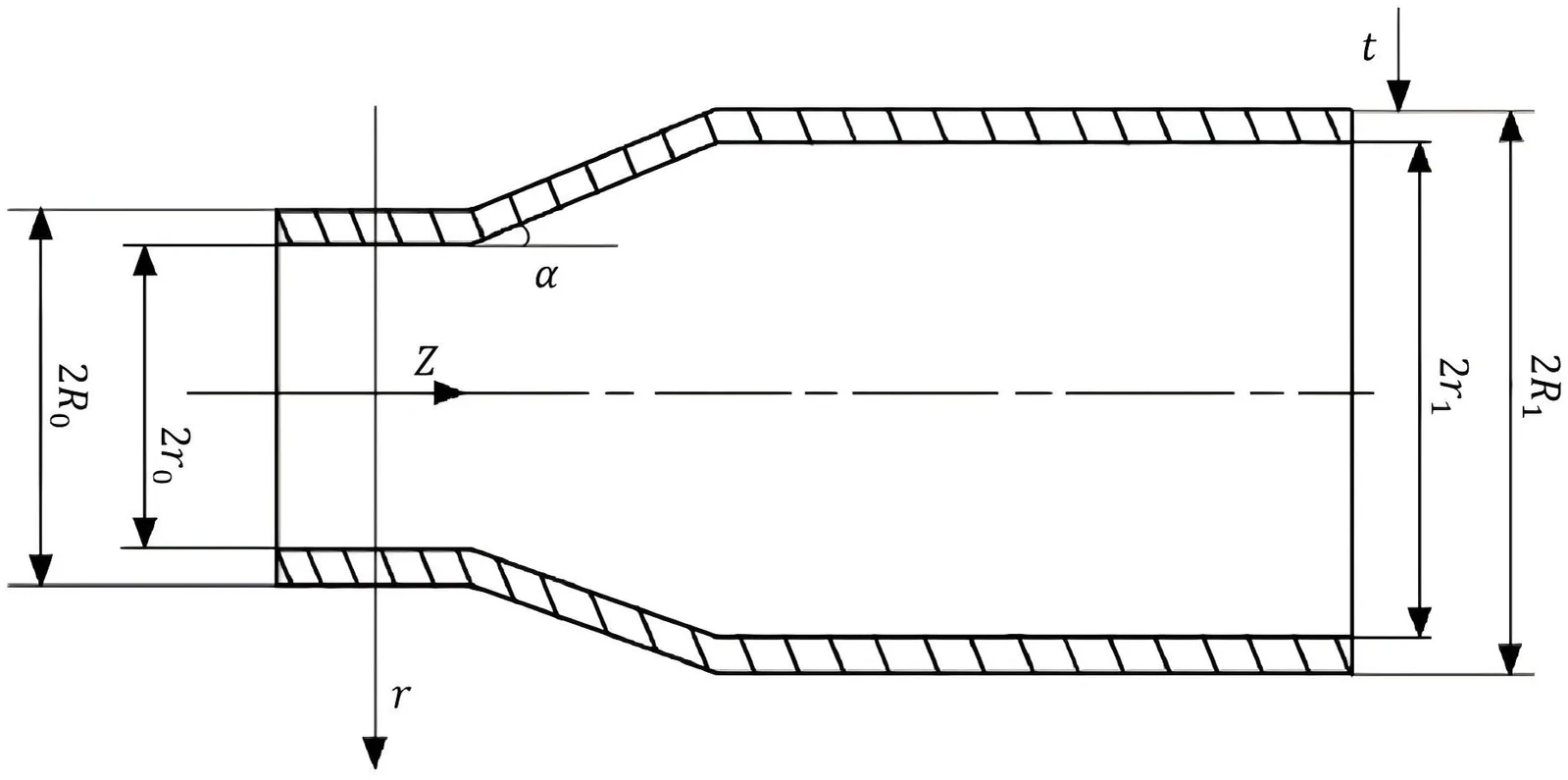
Schematic diagram of dilation deformation.

The stress of the energy-absorbing tube is given as follows [20,21]:


$$\begin{array}{c} \begin{array}{c} \sigma_{z}=\beta \sigma_{s}\frac{a+1}{a}\left[1-{\left(\frac{R_{\text{1}}+r_{\text{1}}}{R_{\text{0}}+r_{\text{0}}}\right)}^{2a}\right] \end{array} \end{array}$$ (1)

where μ is the coefficient of friction between the inner wall of the energy-absorbing tube and the expanded conical sleeve. $\beta =\frac{K}{\sigma_{s}}$, $a=\frac{1+\mu cot\alpha }{1-\mu tan\alpha }-1$.

Based on Equation (1), the bearing capacity of the energy-absorbing tube throughout the deformation process is derived as follows:


$$\begin{array}{c} F_{max}=1.15\pi \left(2r_{\text{0}}t+t^{\text{2}}\right)\sigma_{s}\frac{a+1}{a}\left[1-{\left(\frac{2r_{\text{1}}+t}{2r_{\text{0}}+t}\right)}^{2a}\right] \end{array}$$ (2)

The energy absorbed during the expansion deformation process of the energy-absorbing tube is given as:


$$\begin{array}{c} \begin{array}{c} E=1.15\pi \left(2r_{\text{0}}t+t^{\text{2}}\right)\sigma_{s}\frac{a+1}{a}\left[1-{\left(\frac{2r_{\text{1}}+t}{2r_{\text{0}}+t}\right)}^{2a}\right]\left(L-\frac{r_{\text{1}}-r_{\text{0}}}{sin\alpha }\right) \end{array} \end{array}$$ (3)

where L is the length of the energy-absorbing tube, and δ is the compression displacement of the energy-absorbing tube.

As indicated by Equation (2), the bearing capacity of the energy-absorbing tube throughout its deformation is governed by multiple interrelated parameters, including the cone angle of the expanded conical sleeve, the wall thickness and inner diameter of the tube, the outer diameter at the larger end of the conical sleeve, and the yield strength of the tube material. A parametric study was therefore conducted utilizing MATLAB software.

The effects of the key parameters on the bearing capacity are presented in Fig 5. It can be observed from Fig 5 that excessively small (<10°) or excessively large (>60°) cone angles result in a sharp decrease or a sharp increase in the bearing capacity, respectively, which would compromise the stable functioning of the constant-resistance energy-absorbing mechanism. In contrast, for cone angles ranging from 10° to 60°, the bearing capacity varies in a relatively smooth and controllable manner. The bearing capacity exhibits an approximately linear increase with the enlargement of both the outer diameter at the larger end of the conical sleeve and the tube wall thickness, while it decreases approximately linearly with the increase in the tube inner diameter. Taking into account the bolt rod strength and the properties of the surrounding rock, the bearing capacity of the energy-absorbing device is designed to be slightly greater than the yield strength of the bolt body, thereby preventing premature failure of the energy-absorbing structure prior to the onset of yielding in the bolt rod.

**Fig 5:**
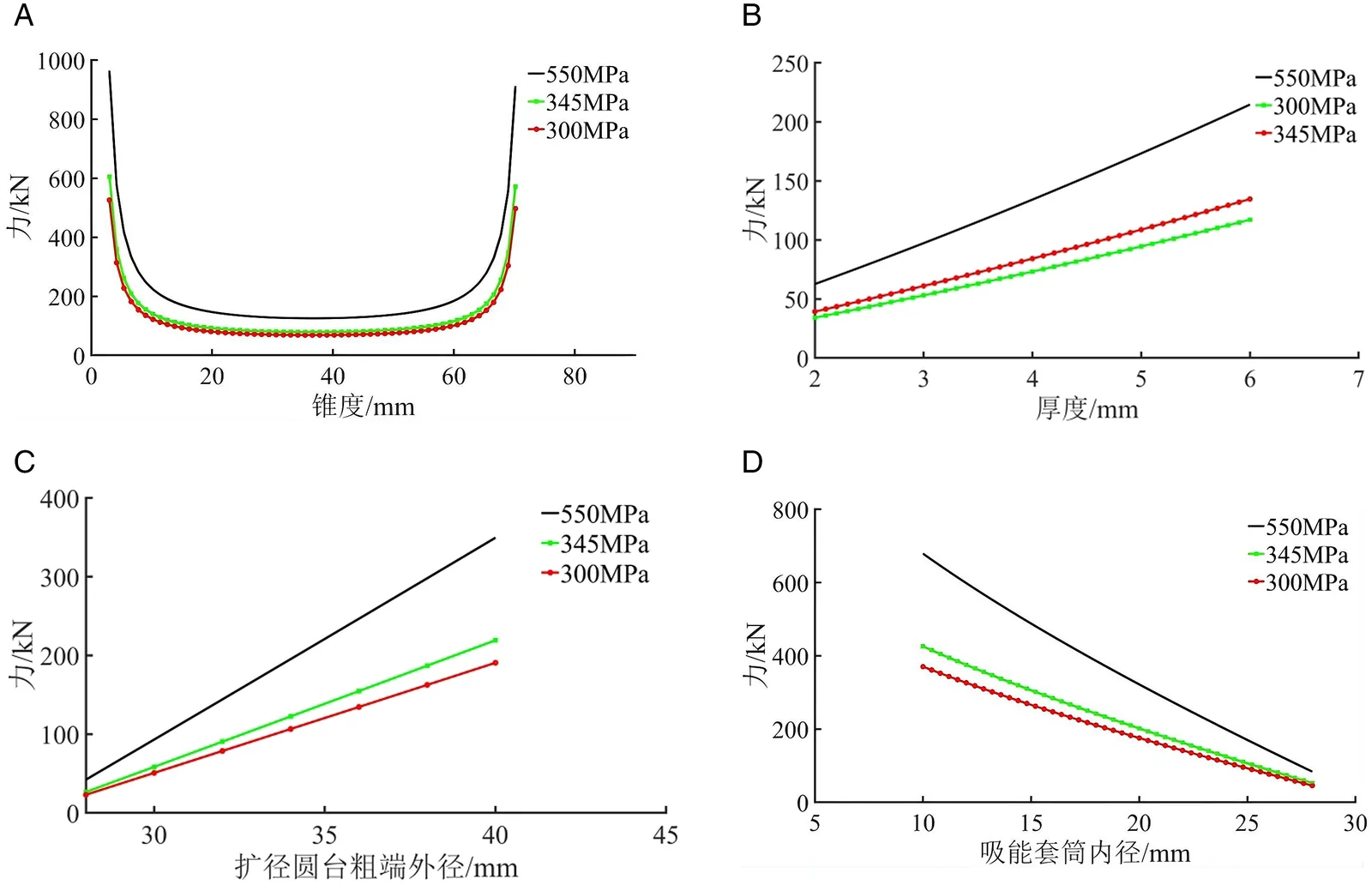
Influence of various parameters on the load-bearing capacity. (a) Bearing capacity versus taper angle curve, (b) Bearing capacity versus wall thickness curve, (c) Bearing capacity versus inner diameter of the energy-absorbing tube, (d) Bearing capacity versus outer diameter of the larger end of the expanded conical sleeve.

### 2.4. Working principle of extended constant-resistance energy-absorbing bolts

Upon installation of the constant-resistance energy-absorbing bolt in the roadway, the axial load imposed on the bolt progressively rises in response to the surrounding rock deformation. The corresponding force–displacement response is depicted in Fig 6, and the entire operational process can be characterized by the following sequential stages:

**Fig 6:**
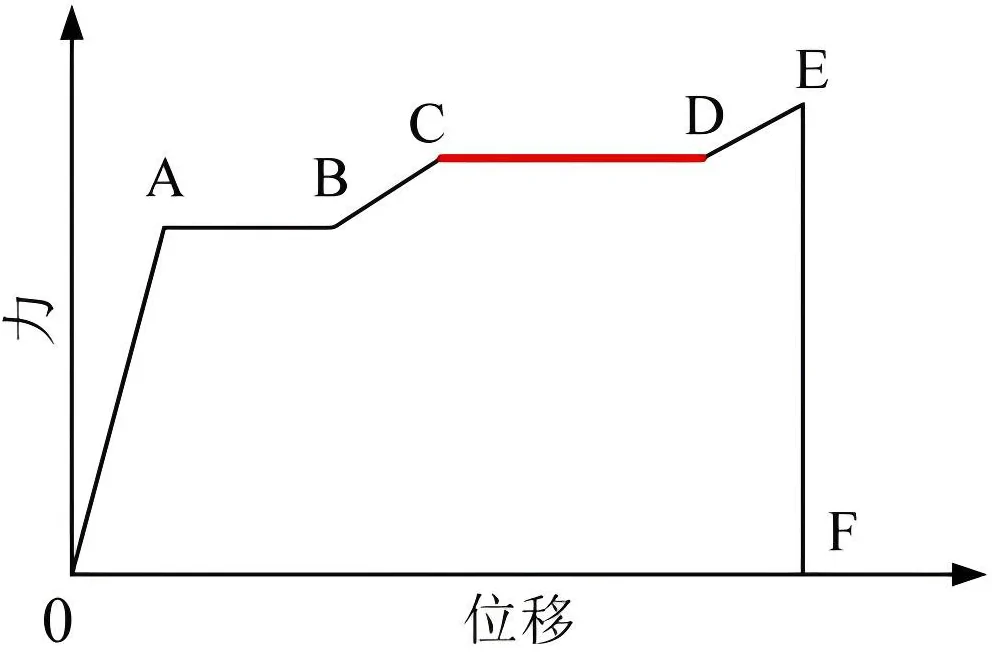
Force–displacement curve of the constant-resistance energy-absorbing bolt.

(1) Elastic deformation stage (OA): The bolt rod undergoes reversible linear elastic deformation, during which the support resistance increases linearly with increasing displacement.
(2) Bolt yielding stage (AB): The axial tensile force remains below the expansion resistance of the energy-absorbing tube; the bolt rod experiences irreversible plastic tensile elongation, whereas the tube stays relatively stationary relative to the rod.
(3) Pre-hardening stage (BC): The bolt material exhibits strain hardening, with the load-bearing capacity continuously rising until the tensile force attains the threshold required to activate the energy-absorbing tube.
(4) Constant-resistance energy-absorbing stage (CD): The expanded conical sleeve induces circumferential expansion plastic deformation in the energy-absorbing tube, accompanied by frictional sliding between the rod and the tube.
(5) Post-hardening stage (DE): Upon the axial tensile force reaching the maximum expansion resistance of the tube, the expansion deformation terminates, and the threaded steel rod re-enters the elastic regime.
(6) Fracture failure stage (EF): Should the surrounding rock deformation persist and the load escalate to the fracture strength of the rod, the threaded steel bar ruptures, resulting in complete failure of the constant-resistance energy-absorbing bolt.

## 3. Analysis of anchor bolt mechanical properties under static loading

### 3.1. Establishment of finite element model for anchor bolts

Finite element models for conventional rock bolts and extended constant-resistance energy-absorbing rock bolts were established using ABAQUS finite element software. The main structure of the conventional anchor model comprises three components: the rod body, the tray, and the nut. The rod body has a diameter of 20 mm and a total length of 2500 mm. The tray has a planar dimension of 150 mm × 150 mm, a thickness of 10 mm, and a central circular hole with a diameter of 25 mm. The nut is square-shaped with a side length of 20 mm and a thickness of 13 mm. The specific structure of this model is shown in Fig 7. The extended constant-resistance energy-absorbing anchor rod primarily consists of a rod body, a tray, an energy-absorbing tube, an expanded tapered section, and a nut: the rod body has a length of 2500 mm and a diameter of 22 mm; The tray dimensions are 200 × 200 × 10 mm; the energy-absorbing tube has an inner diameter of 26 mm and a wall thickness of 4.2 mm; the tapered expansion cone has a 22.5° angle and a height of 12 mm, with the narrow end diameter matching the rod body (22 mm) and the wide end diameter being 32 mm; the energy-absorbing tube is designed with a displacement distance of 280 mm, as illustrated in Fig 8.

**Fig 7:**
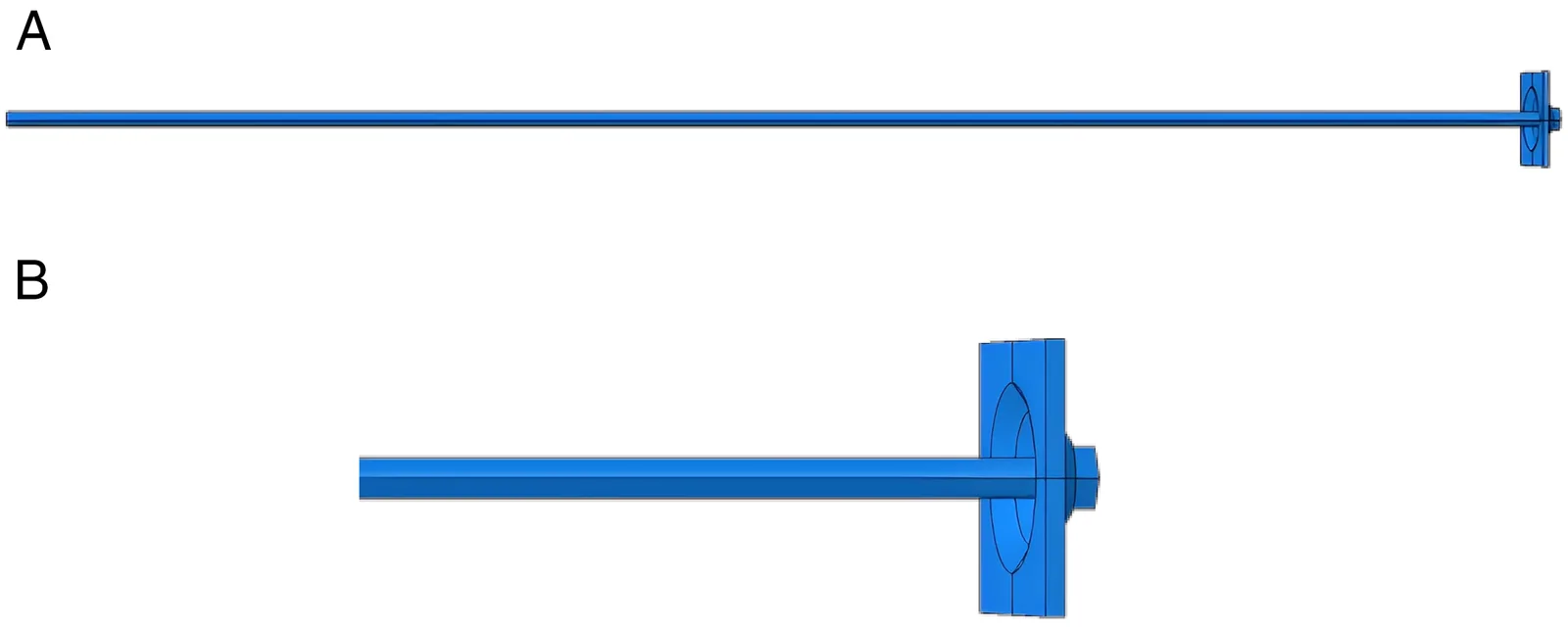
Conventional bolt model. (a) Conventional bolt, (b) Bolt tail.

**Fig 8:**
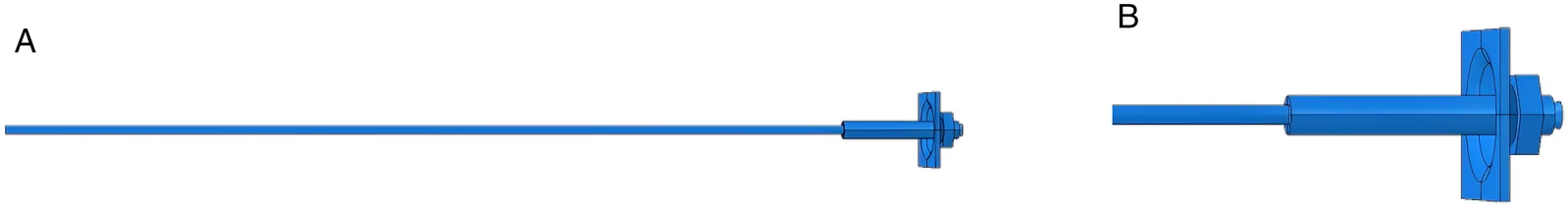
Constant-resistance energy-absorbing bolt model. (a) Elongated energy-absorbing rock bolt with constant resistance, (b) Tail of the elongated energy-absorbing rock bolt.

The analysis step employs dynamic display simulation of the tensile process. Both conventional anchor rods and extended constant-resistance energy-absorbing anchor rods utilize Q345 low-alloy high-strength steel for their shafts. while the nuts are made of Q400 low-alloy high-strength steel. The trays and energy-absorbing sleeves of the extended constant-resistance energy-absorbing bolts are fabricated from Q550 low-alloy high-strength steel, and the tapered expansion section of the extended constant-resistance energy-absorbing bolts is constructed from Q700 low-alloy high-strength steel. Material properties are detailed in Table 3. Additionally, the threaded steel rod body employs a flexible damage criterion, with the element deletion option selected in the assigned element type.

**Table 3:** Material properties.

Material	Density/ (g·cm^−3^)	Elastic Modulus/ MPa	Yield Strength/ MPa	Poisson’s Ratio
Q345	7.85	206000	345	0.30
Q400	7.85	206000	400	0.30
Q550	7.85	210000	550	0.35
Q700	7.85	206000	700	0.30

To verify the stability of the finite element simulation results, a mesh convergence analysis was conducted on the static tensile model of the bolt. The computational results indicate that as the element size is gradually reduced from 6 mm to 4 mm, the bearing capacity and elongation obtained from the model tend to stabilize. When the element size is further refined to below 4 mm, the variation in key mechanical indices is less than 2%, satisfying the convergence criterion; however, the computational cost increases significantly. Therefore, the mesh element size for the bolt body was set to 4 mm, while the bearing plate and nut were meshed with an element size of 6 mm. The seeding constraint was set to prohibit alteration of the number of elements. The element type selected was the eight-node linear hexahedral element with incompatible modes (C3D8I), and the element deletion function was activated to simulate element failure behavior during the material failure process.

The anchor rod employs general contact globally, with surface-to-surface contact applied in critical monitoring areas (top and bottom surfaces of the tray). A penalty function is used with a friction coefficient of 0.3. The rod’s left end is subjected to a fully fixed constraint, while the nut end permits only axial displacement. All components are connected via binding constraints.

Under static loading, a 650 mm displacement load is applied from the right to the left side of the perforated discrete rigid plate attached to the pallet. The central hole diameter of this rigid plate exceeds the anchor rod shaft diameter. Under impact loading, a perforated rigid disk strikes the anchor rod from the right to the left side of the pallet at varying impact energies and velocities to analyze its dynamic response characteristics.

### 3.2. Conventional anchor bolt static tensile testing and finite element validation

Conventional quasi-static tensile tests on anchor bolts were conducted using a LAW-600 microcomputer-controlled electric tensile testing machine, with a tensile rate set at 50 mm/min. The test setup is shown in Fig 9.

**Fig 9:**
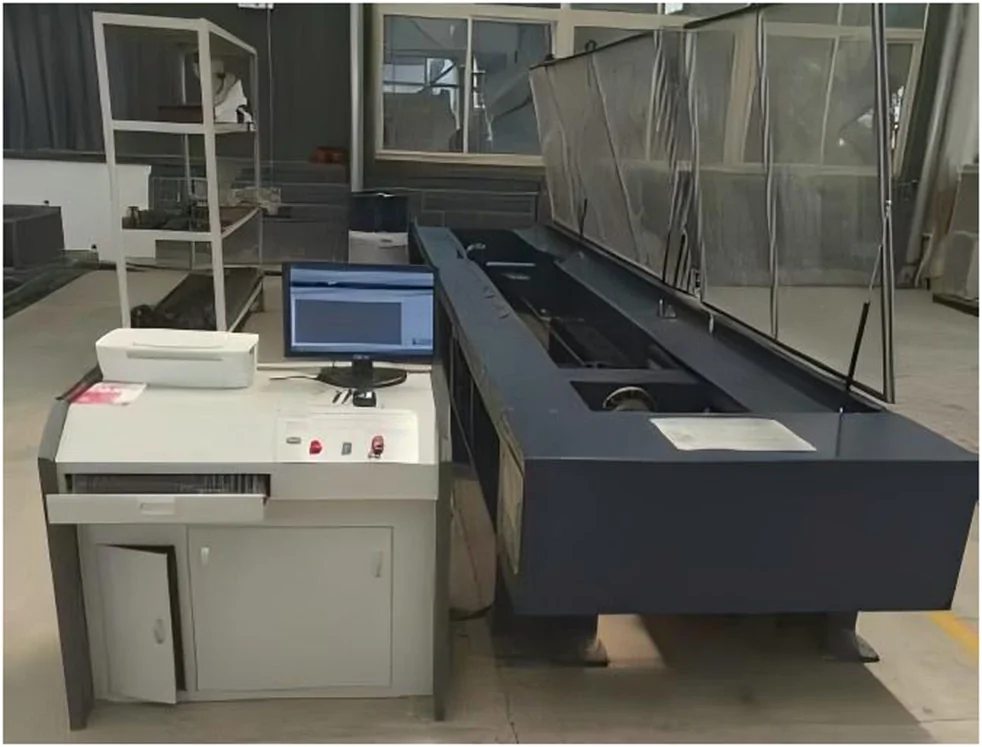
Experimental devices.

Comparing conventional anchor rod static tensile tests with numerical simulation data, the specific mechanical properties are summarized in Table 4. The anchor rod force-displacement curve is shown in Fig 10, while the energy absorption-displacement curve is depicted in Fig 11.

**Table 4:** Comparison of mechanical properties between experimental and simulated results for the conventional bolt.

Mechanical parameter	Experimental data	Simulated data	Error/ %
Yield-stage displacement/ mm	5.12	4.97	2.93
Fracture displacement/ mm	222.88	225.61	1.22
Elongation/ %	8.92	9.02	1.12
Fracture load/ kN	158.34	162.83	2.84
Total energy absorption/ kJ	30.64	31.64	3.26

**Fig 10:**
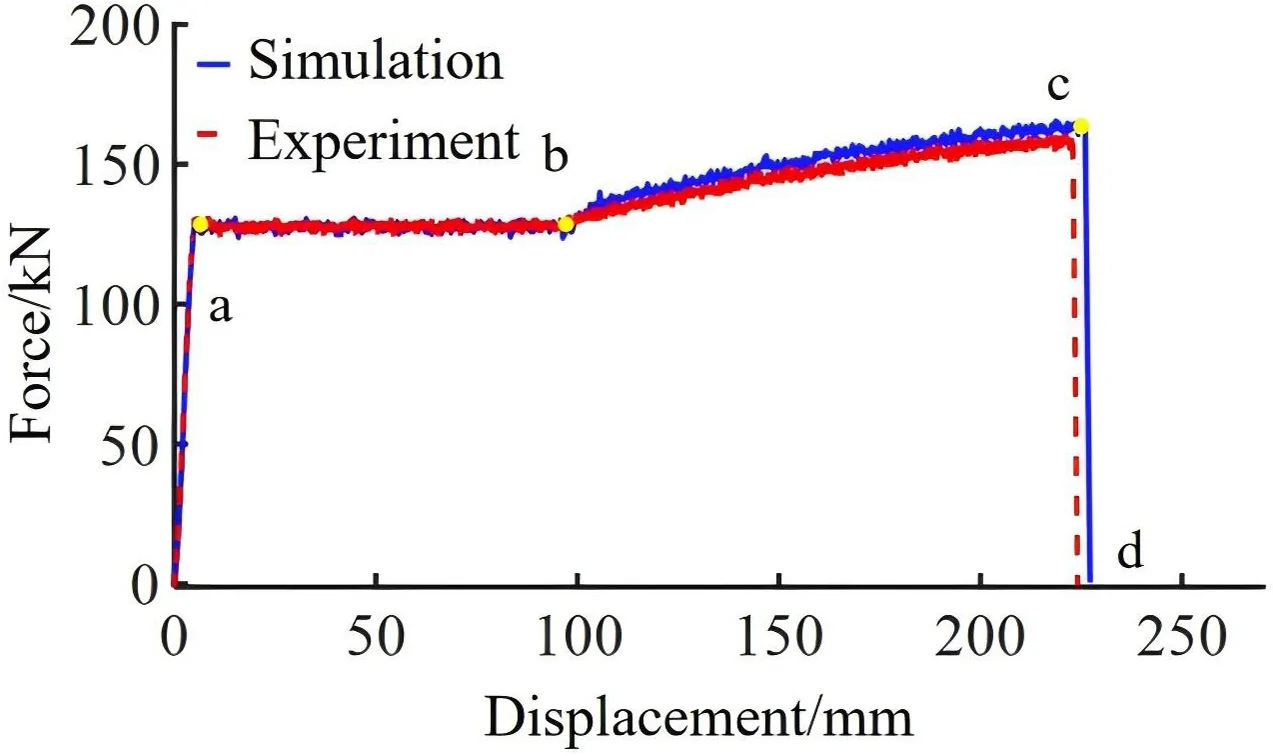
Bolt force-displacement curve.

**Fig 11:**
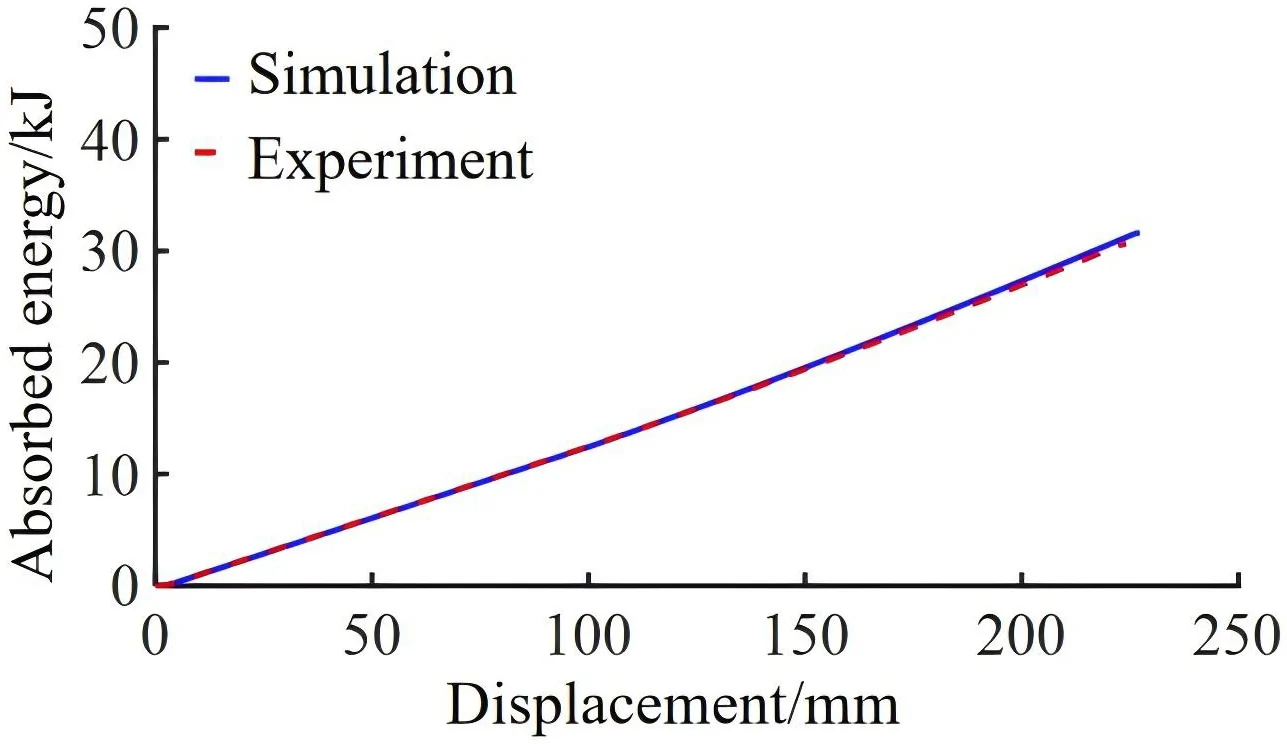
Bolt energy absorption-displacement curve.

Based on the comparison of experimental and numerical simulation data: – Yield stage displacements were 5.12 mm and 4.97 mm, differing by 0.15 mm;Failure displacements were 222.88 mm and 225.61 mm, differing by 2.73 mm; – Elongation rates were 8.92% and 9.02%, differing by 0.1%; The fracture loads were 158.34 kN and 162.83 kN, differing by 4.49 kN; the total energies were 30.64 kJ and 31.64 kJ, differing by 1 kJ. Considering the consistency in curve shapes between Fig 9 and Fig 10, the consistency in curve morphology indicates that under conventional static tensile conditions for anchor rods, the experimental results exhibit an extremely high degree of agreement with the numerical simulation results. In summary, under conventional static tensile loading of anchor rods, the experimental results exhibit a high degree of similarity with the numerical simulation results. The numerical simulation method employed demonstrates good feasibility and effectiveness in anchor rod tensile studies.

### 3.3. Static tensile simulation of extended constant-resistance energy-absorbing anchor rods

The force–displacement responses of the two bolt types are presented in Fig 12. It can be observed that, in contrast to the conventional bolt, the constant-resistance energy-absorbing bolt incorporates an additional displacement-compensation stage provided by the energy-absorbing tube during the early hardening phase of the bolt rod. As a result, both the yielding stroke and the total energy absorption of the constant-resistance energy-absorbing bolt are substantially increased relative to those of the conventional bolt.

**Fig 12:**
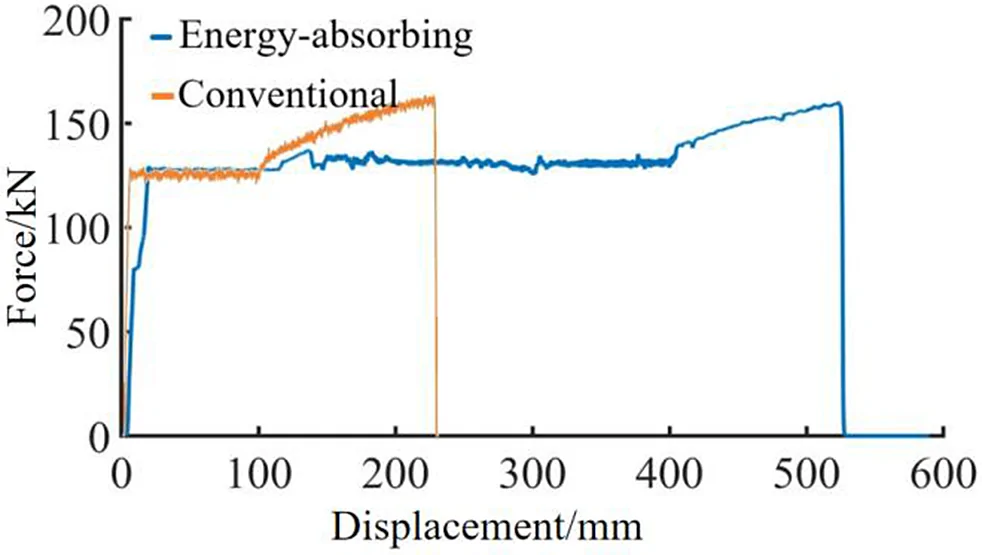
Force-displacement curve.

The breaking load of the conventional anchor rod was 163.9 kN, while that of the extended constant-resistance energy-absorbing anchor rod was 160.6 kN. The close numerical values indicate that the ultimate fracture resistance of both anchor rod types is essentially equivalent. Additionally, during the energy-absorbing deformation of the energy absorber, the extended constant-resistance energy-absorbing anchor rod consistently provided a load-bearing capacity range of 128.2–135.0 kN, demonstrating that the energy-absorbing sleeve maintains a certain load-bearing capacity during the deformation stage.

The comparison of mechanical properties between the constant-resistance energy-absorbing bolt and the conventional bolt under static loading is presented in Table 5. The conventional anchor rod exhibited a breaking displacement of 222.3 mm and an elongation rate of 8.89%, while the extended constant-resistance energy-absorbing anchor rod achieved a breaking displacement of 523.2 mm and an elongation rate increased to 20.93%. The elongation of the extended constant-resistance energy-absorbing anchor rod is 2.35 times that of the conventional anchor rod. Its deformation adaptability significantly outperforms the conventional anchor rod, effectively reducing premature failure caused by insufficient deformation.

**Table 5:** Comparison of static tensile mechanical properties between the two bolt types.

Parameter	Conventional bolt	Constant-resistance energy-absorbing bolt	Ratio
Fracture load/kN	163.9	160.6	1.00
Fracture displacement/mm	222.3	523.2	2.35
Elongation/%	8.89	20.93	2.35
Total energy absorption/kJ	30.9	69.7	2.26

The energy absorption curve is shown in Fig 13. The energy absorption capacity of conventional rock bolts is 30.9 kJ, while that of extended constant-resistance energy-absorbing rock bolts is 69.7 kJ. The energy absorption capacity of extended constant-resistance energy-absorbing rock bolts is 2.26 times that of conventional rock bolts. Through deformation dissipation within the energy absorber structure, the rock bolts’ capacity to absorb impact energy can be significantly enhanced.

**Fig 13:**
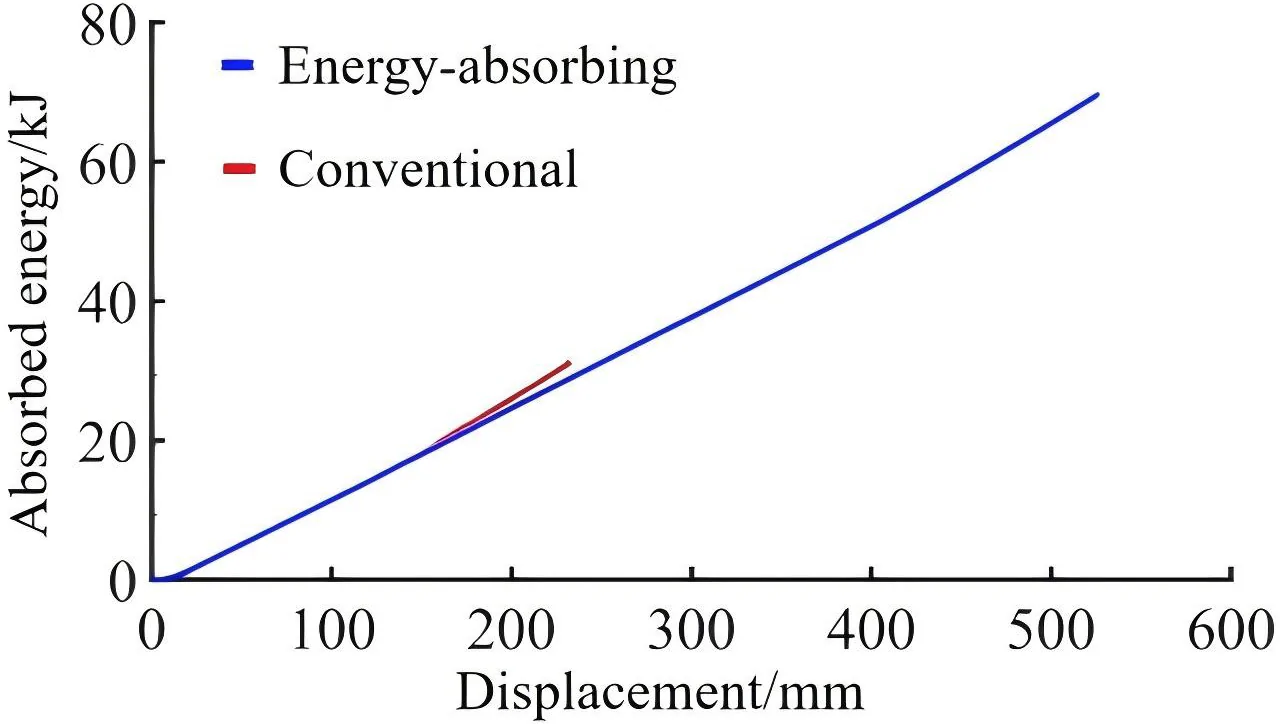
Energy absorption-displacement curve.

## 4. Analysis of mechanical properties of anchor rods under dynamic loading

### 4.1. Calculation cases setup

To investigate the effects of impact energy and impact velocity on the mechanical properties of constant-resistance energy-absorbing bolts and conventional bolts, a total of 12 numerical simulation cases were designed, as shown in Table 6.

**Table 6:** Calculation cases.

Parameter	Value
Impact energy/ kJ	20, 50, 90
Impact velocity/ (m·s^−1^)	3, 5, 7, 9
Bolt type	Constant-resistance energy-absorbing bolt, Conventional bolt

(1) Impact energy conditions: Three impact energy levels, namely 20 kJ, 50 kJ, and 90 kJ, were defined based on the relative relationship between the applied impact energy and the energy absorption capacities of the two bolt types. These levels correspond to three representative scenarios: impact energy lower than the absorption capacity of both bolt types (20 kJ), intermediate between the capacity of the conventional bolt and that of the constant-resistance energy-absorbing bolt (50 kJ), and higher than the capacity of both bolt types (90 kJ).
(2) Impact velocity conditions: With the impact energy fixed at 90 kJ, at which both bolt types are expected to fracture, four impact velocities of 3 m/s, 5 m/s, 7 m/s, and 9 m/s were prescribed.

All finite element model parameters for both bolt types, with the exception of the loading conditions, were kept identical to those employed in the static simulations. A rigid disc of 10 mm in thickness, featuring a central hole slightly larger than the diameter of the steel connector, was used as the impactor. Impact loads were applied from the right side of the bearing plate along the negative Z-axis direction at the designated velocities until fracture of the bolt occurred.

### 4.2. Numerical analysis of bolt mechanical performance under impact energy

Fig 14 shows the stress distribution map during tensile deformation of conventional anchor rods and extended constant-resistance energy-absorbing anchor rods subjected to a 90 kJ impact. Both anchor rod types fractured under the 90 kJ energy impact, indicating that impact energy exceeding their design load limits causes irreversible material failure. Under a 50 kJ energy impact, the conventional anchor rod fractured, while the extended constant-resistance energy-absorbing anchor rod maintained integrity without fracture. This demonstrates that the extended constant-resistance energy-absorbing anchor rod can withstand higher energy input under identical impact load conditions, exhibiting superior impact resistance. Under a 20 kJ energy impact, both conventional anchor rods and extended constant-resistance energy-absorbing anchor rods remained intact without fracture. This demonstrates sufficient strength and elongation capacity under mild impact loads.

**Fig 14:**
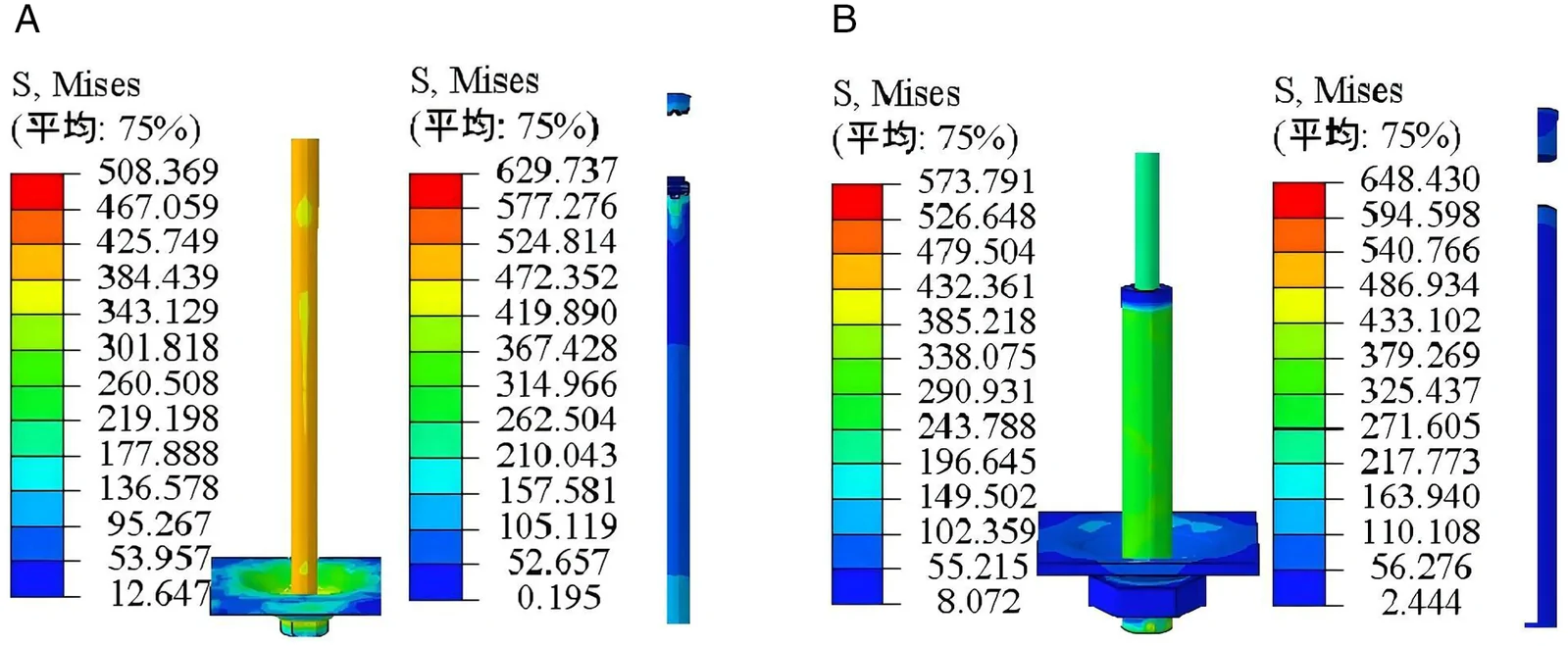
Stress contour diagram of bolt shaft and tail under 90 kJ impact. (a) Conventional rock bolt, (b) Elongated energy-absorbing bolt with constant resistance.

The force–time curves and energy absorption curves of the constant-resistance energy-absorbing bolt and the conventional bolt under impact energies of 20, 50, and 90 kJ are shown in Figs 15–17, respectively. The mechanical properties of the bolts are summarized in Table 7. From Figs 15–17 and Table 7, the following observations can be made:

**Table 7:** Bolt mechanical properties.

Anchor Bolt Type	Impact Energy/ kJ	Impact Velocity/ m·s^−1^	Fracture Status	Fracture Load/ kN	Fracture Displacement/ mm	Elongation/ %
Conventional	20	7	Intact	–	–	–
energy-absorbing	20	7	Intact	–	–	–
Conventional	50	7	Fractured	164.1	226.9	9.08
energy-absorbing	50	7	Intact	–	–	–
Conventional	90	7	Fractured	162.0	225.1	9.00
energy-absorbing	90	7	Fractured	160.7	513.4	20.5

**Fig 15:**
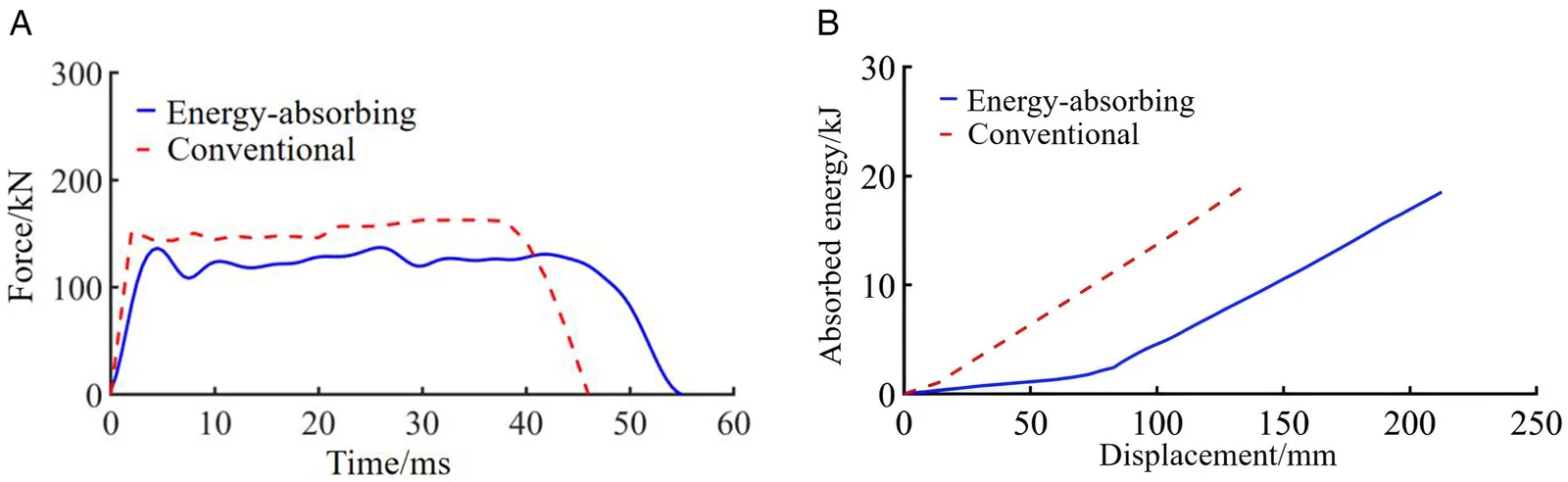
Force-time and energy absorption curves of the two bolts under 20 kJ impact. (a) Force-time curve, (b) Energy absorption curve.

**Fig 16:**
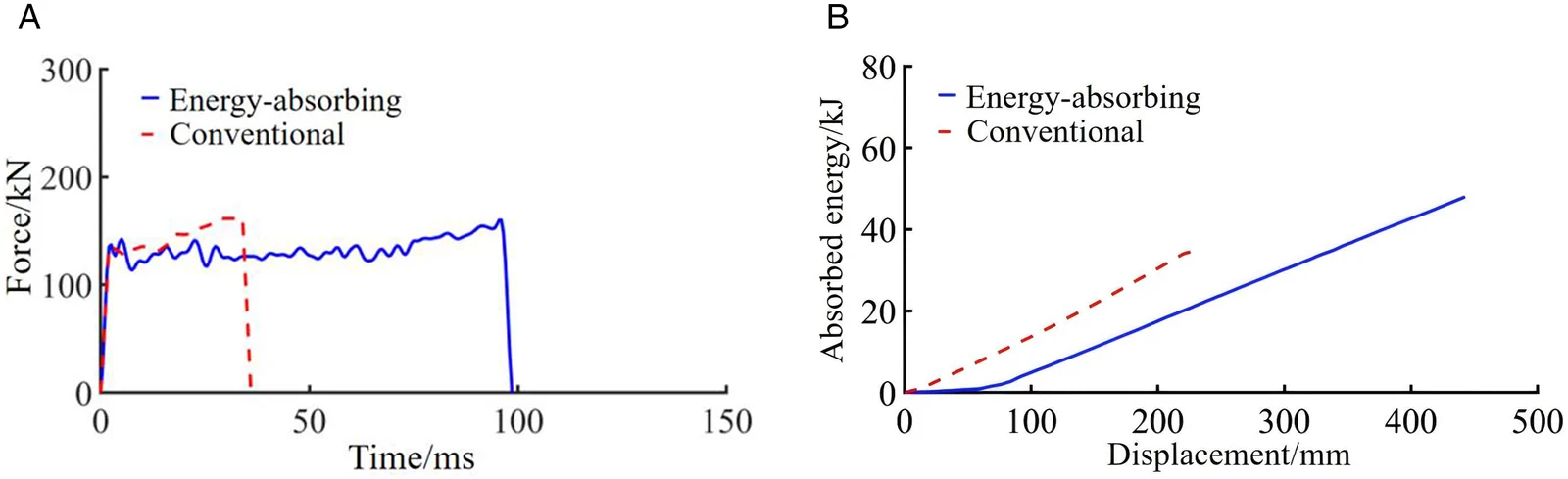
Force-time and energy absorption curves of the two bolts under 50 kJ impact. (a) Force-time curve, (b) Energy absorption curve.

**Fig 17:**
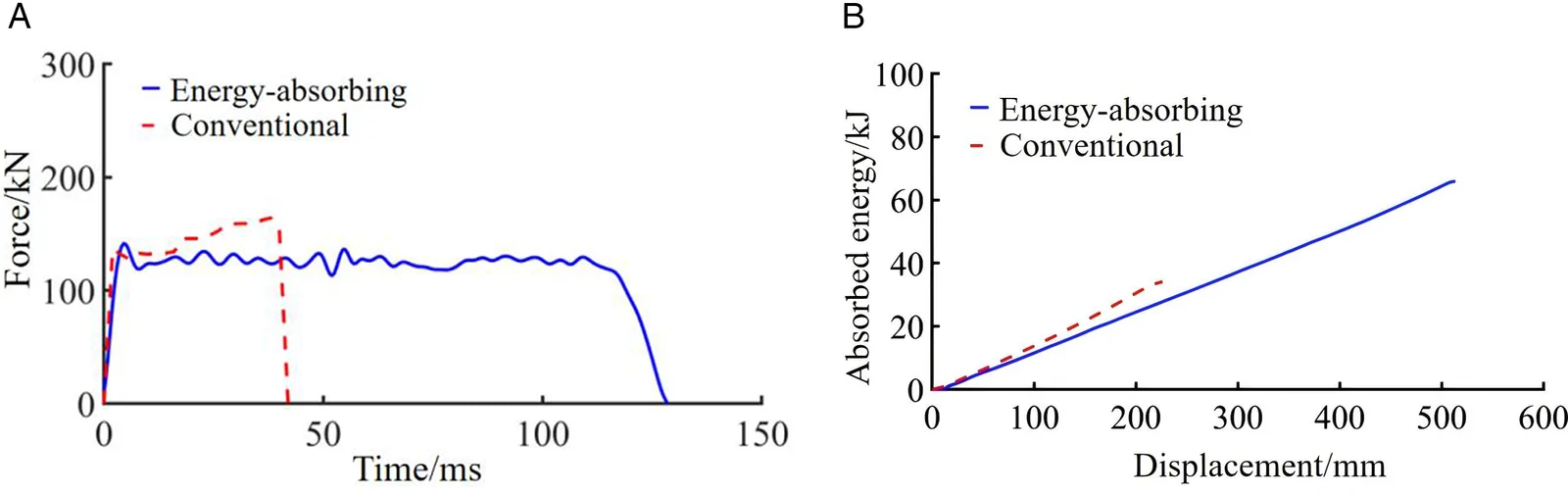
Force-time and energy absorption curves of the two bolts under 90 kJ impact. (a) Force-time curve, (b) Energy absorption curve.

(1) Under dynamic loading, the extended constant-resistance energy-absorbing anchor rod exhibits more stable repeatable deformation characteristics. Its energy absorber maintains a constant load-bearing range of 124.8–133.8 kN during impact, demonstrating a stable deformation threshold and excellent impact resistance.
(2) Under static loads and impact loads of 50 kJ and 90 kJ, the conventional anchor rods exhibited breaking loads of 163.9 kN, 164.1 kN, and 162.0 kN, respectively; breaking displacements of 222.3 mm, 226.9 mm, and 225.1 mm, respectively; elongation rates of 8.89%, 9.08%, and 9.00%, respectively; and absorbed energies of 30.9 kJ, 34.7 kJ, and 34.1 kJ, respectively. Under impact loading, the breaking load, breaking displacement, elongation rate, and absorbed energy of conventional bolts showed no significant changes compared to static loading conditions, demonstrating consistent mechanical properties.
(3) Under an impact energy of 20 kJ, the elongated constant-resistance energy-absorbing bolt underwent tensile yielding of the bolt body only, whereas the energy-absorbing tube exhibited no apparent deformation. When the impact energy was increased to 50 kJ, the reaction force reached the deformation threshold of the energy-absorbing tube, triggering its diameter-expansion deformation. Under both static loading and 90 kJ impact loading, the energy-absorbing tube of the elongated constant-resistance energy-absorbing bolt was fully deformed, accompanied by fracture of the bolt body. Under static loading, the fracture load, fracture displacement, elongation, and energy absorption were 160.6 kN, 523.2 mm, 20.9%, and 69.7 kJ, respectively; under 90 kJ impact loading, the corresponding values were 160.1 kN, 513.4 mm, 20.5%, and 65.9 kJ. Compared with the static loading condition, the impact loading condition resulted in moderate reductions in fracture displacement, elongation, and energy absorption, whereas the fracture load remained essentially unchanged.
(4) Under a 90 kJ impact load, significant differences emerged in the mechanical properties between conventional anchor rods and extended constant-resistance energy-absorbing anchor rods. Regarding breaking load, the conventional anchor rod exhibited 162.0 kN, while the extended constant-resistance energy-absorbing anchor rod reached 160.1 kN, indicating essentially equivalent ultimate breaking load capacities for both types. Regarding breaking displacement, conventional bolts recorded 225.1 mm, while extended constant-resistance energy-absorbing bolts reached 513.4 mm—2.28 times that of conventional bolts. In terms of elongation, conventional bolts exhibited 9.00%, whereas extended constant-resistance energy-absorbing bolts demonstrated 20.5%—also 2.28 times higher than conventional bolts. In summary, under high-energy impact conditions, although the elongated constant-resistance energy-absorbing anchor rod exhibits a fracture load similar to that of the conventional anchor rod, it demonstrates significant advantages in fracture displacement, elongation rate, and energy absorption capacity. Its energy absorption capability and impact resistance far surpass those of the conventional anchor rod, indicating significantly greater application potential in complex stress conditions.

### 4.3. Analysis of impact velocity effects

The mechanical properties of both bolt types under different impact velocities are summarized in Table 8. Under different impact velocity conditions, the force-time curves for conventional anchor rods and extended constant-resistance energy-absorbing anchor rods are shown in Figs 18 and 19, respectively. At impact velocities of 3 m/s, 5 m/s, 7 m/s, and 9 m/s, the fracture loads of conventional anchor rods were 161.4 kN, 161.5 kN, 160.3 kN, and 161.6 kN respectively, with an average value of 161.2 kN. The breaking loads for the extended constant-resistance energy-absorbing anchor rods were 161.1 kN, 160.3 kN, 162.6 kN, and 160.3 kN, respectively, with an average of 161.1 kN. As the impact velocity increased, the breaking loads for both types of anchor rods remained essentially stable. Regarding impact resistance time, conventional anchor rods recorded 80.2 ms, 48.7 ms, 36.5 ms, and 26.1 ms; while the extended constant-resistance energy-absorbing anchors recorded 190.6 ms, 134.8 ms, 94.5 ms, and 75.2 ms, respectively—2.38 times, 2.77 times, 2.59 times, and 2.88 times longer than conventional anchors. The impact resistance time for both anchor types exhibited a nonlinear decrease with increasing impact velocity. However, at the same impact velocity, the extended constant-resistance energy-absorbing anchors demonstrated longer impact resistance times.

**Table 8:** The mechanical behaviors of traditional and constant – resistance energy – absorbing bolts under various impact velocities.

Bolt type	Impact velocity (m·s^−1^)	Fracture load (kN)	Fracture displacement (mm)	Elongation (%)	Energy absorption (kJ)	Impact resistance duration (ms)	Energy absorption rate (J·ms^−1^)
Conventional bolt	3	161.4	228.1	9.1	32.5	80.2	405.2
Conventional bolt	5	161.5	216.8	8.7	30.7	48.7	630.4
Conventional bolt	7	160.3	214.2	8.6	30.2	36.5	827.4
Conventional bolt	9	161.6	197.9	7.9	28.5	26.1	1092.0
Elongated constant-resistance energy-absorbing bolt	3	161.1	525.2	21.0	67.4	190.6	353.6
Elongated constant-resistance energy-absorbing bolt	5	160.3	532.8	21.3	66.6	134.8	494.1
Elongated constant-resistance energy-absorbing bolt	7	162.6	526.2	21.0	66.3	94.5	701.6
Elongated constant-resistance energy-absorbing bolt	9	160.3	533.9	21.4	67.3	75.2	894.9

**Fig 18:**
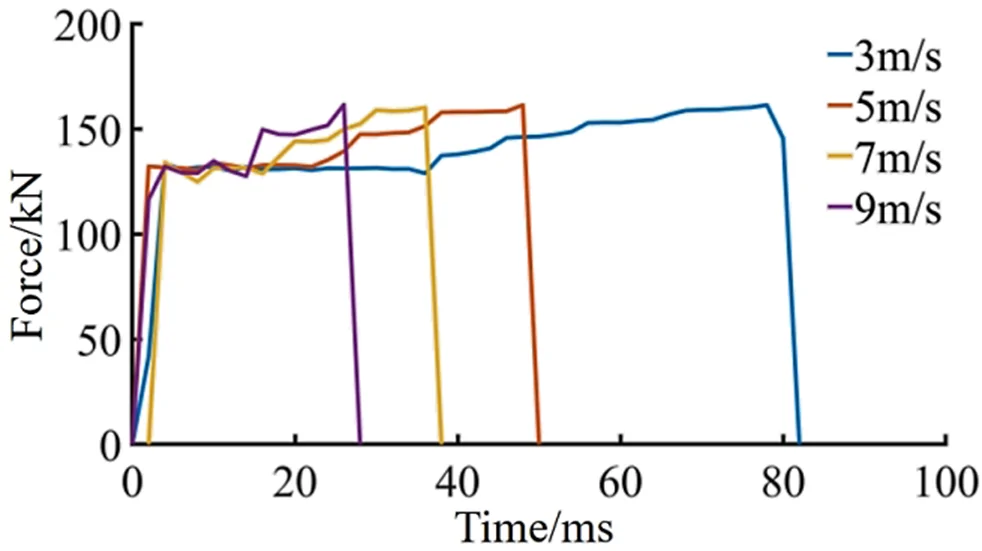
Force-time curves of conventional bolts.

**Fig 19:**
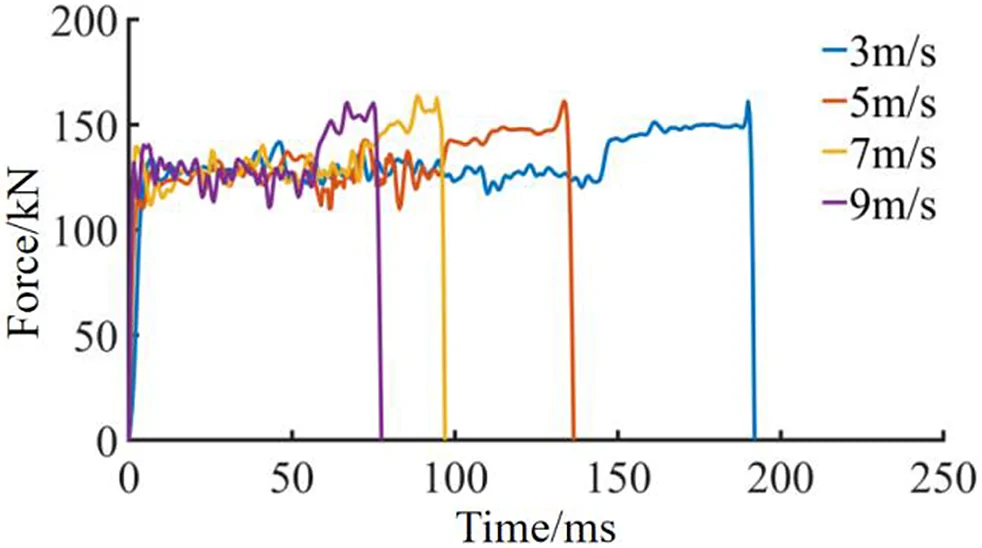
Force-time curves of constant-resistance energy-absorbing bolts.

The energy absorption-displacement curves for conventional anchor rods and extended constant-resistance energy-absorbing anchor rods under different impact velocities are shown in Figs 20 and 21, respectively. At impact velocities of 3 m/s, 5 m/s, 7 m/s, and 9 m/s, the fracture displacements of conventional bolts were 228.1 mm, 216.8 mm, 214.2 mm, and 197.9 mm, respectively, with an average of 214.3 mm. The elongation rates were 9.1%, 8.7%, 8.6%, and 7.9%, respectively, with an average of 8.58%. The energy absorption values were 32.5 kJ, 30.7 kJ, 30.2 kJ, and 28.5 kJ, respectively, averaging 30.48 kJ. As the impact velocity increased, the fracture displacement, elongation rate, and energy absorption all showed a gradual decreasing trend. Under impact loading, the yield strength of the threaded steel bars slightly increased, but their plastic deformation capacity significantly weakened, leading to brittle fracture before the bars could fully utilize their plastic deformation properties.

**Fig 20:**
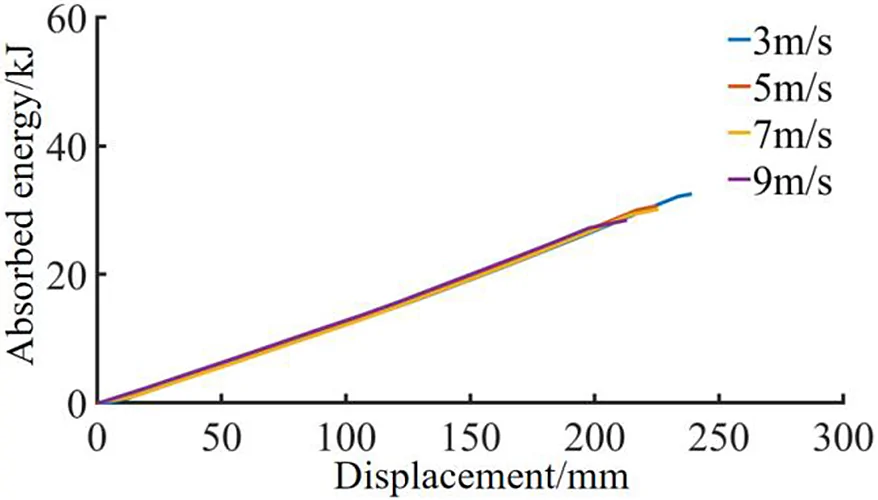
Energy absorption-displacement curve of conventional bolt.

**Fig 21:**
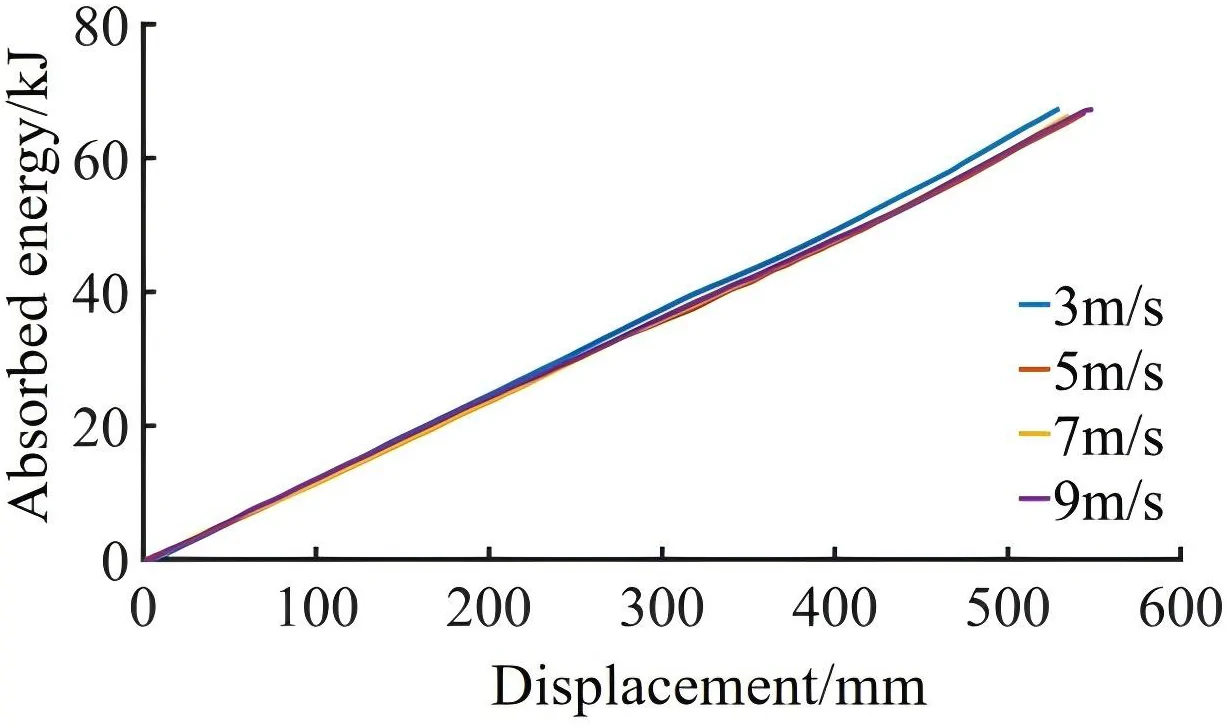
Energy absorption-displacement curve of the constant-resistance energy-absorbing bolt.

The fracture displacements of the extended constant-resistance energy-absorbing anchor rods were 525.2 mm, 532.8 mm, 526.2 mm, and 533.9 mm, respectively, with an average of 529.5 mm. The elongation rates were 21.0%, 21.3%, 21.0%, and 21.4%, respectively, averaging 21.2%. The absorbed energies were 67.4 kJ, 66.6 kJ, 66.3 kJ, and 67.3 kJ, respectively, with an average of 66.9 kJ. When the impact velocity increased from 3 m/s to 9 m/s, the breaking load, elongation, and absorbed energy showed no significant changes, with only slight fluctuations in the breaking displacement. This indicates that the impact velocity has a minor effect on their mechanical properties. Impact energy was primarily dissipated through plastic flow in the energy-absorbing tube, significantly reducing the strain rate experienced by the rod body and thereby markedly weakening the strain rate hardening effect.

The extended constant-resistance energy-absorbing anchor rod outperformed conventional anchor rods in terms of breaking displacement, elongation, and energy absorption, with average values 2.47 times, 2.47 times, and 2.19 times those of conventional anchor rods, respectively. With higher elongation and breakage displacement, these bolts better accommodate large rock mass deformations caused by impact loads, preventing premature failure. Simultaneously, their superior energy absorption capacity helps dissipate impact energy, reducing damage to both the rock mass and support structures.

At impact velocities of 3 m/s, 5 m/s, 7 m/s, and 9 m/s, the energy absorption rates were 353.4 J/ms, 494.1 J/ms, 701.6 J/ms, and 894.9 J/ms, respectively, exhibiting a significant nonlinear increase with rising impact velocity. This indicates the anchor rod can absorb greater energy per unit time, facilitating rapid energy dissipation during impacts. This mitigates instantaneous stresses on the support system and surrounding rock, thereby enhancing roadway stability and safety. The nonlinear increase in energy absorption rate stems from its unique structural design, demonstrating excellent dynamic response characteristics. This further validates its superior impact resistance compared to conventional anchor rods.

## 5. Conclusions

To address the issue of premature anchor failure caused by mismatched clearance distances between energy-absorbing rock bolts and hydraulic supports, an extended constant-resistance energy-absorbing rock bolt with a longer clearance distance was designed for improved coordination with hydraulic supports. The impact resistance performance of this extended bolt under static loads, varying impact energies, and impact velocities was investigated, yielding the following primary conclusions:

(1) Based on the yield load (135.33 kN) and breaking load (163.9 kN) of conventional bolts, the load-bearing range of the energy-absorbing tube was determined to be 128.2–135.0 kN. Accordingly, its specifications were set as follows: inner diameter 26 mm, wall thickness 4.2 mm, height 300 mm; The tapered section height is 12 mm, with a tapered end diameter of 22 mm, a coarse end diameter of 32 mm, and a bottom cone angle of 22.5°.
(2) Under static loading, the elongated constant-resistance energy-absorbing anchor rod exhibited a breaking load of 160.6 kN, breaking displacement of 523.2 mm, elongation of 20.93%, and energy absorption of 69.7 kJ. The elongation and energy absorption of the extended constant-resistance energy-absorbing anchor rod were 2.35 times and 2.26 times that of conventional anchor rods, respectively. Under 90 kJ impact energy, these values were 2.28 times and 1.93 times that of conventional anchor rods, respectively. At different impact velocities, the average elongation and energy absorption were 2.47 times and 2.19 times that of conventional anchor rods, respectively. By extending the displacement distance, the extended constant-resistance energy-absorbing anchor rod achieves coordinated support with hydraulic supports, preventing premature failure caused by mismatched displacement.
(3) The constant-resistance energy-absorbing bolt showed significantly better mechanical performance than the conventional bolt under both static and impact loads. The structural sliding deformation of its energy-absorbing device effectively extended the yield distance and greatly improved deformation capacity and energy absorption efficiency. At impact velocities up to 9 m/s, the fracture load, elongation, and energy absorption of the bolt varied little, whereas the impact resistance duration decreased as the impact velocity increased.
